# Increased background parenchymal enhancement on peri-menopausal breast magnetic resonance imaging

**DOI:** 10.1016/j.ejro.2024.100611

**Published:** 2024-11-18

**Authors:** Hidemi Okuma, Amro Masarwah, Aleksandr Istomin, Aki Nykänen, Juhana Hakumäki, Ritva Vanninen, Mazen Sudah

**Affiliations:** a Institute of Clinical Medicine, School of Medicine, Clinical Radiology, University of Eastern Finland, P.O. Box 1627, Kuopio Fl 70211, Finland; b Department of Clinical Radiology, Diagnostic Imaging Center, Kuopio University Hospital, P.O. Box 100, Kuopio Fl 70029, Finland

**Keywords:** Breast MRI, Background parenchymal enhancement, Breast cancer, Menopausal transition, Perimenopause

## Abstract

**Objectives:**

To examine the background parenchymal enhancement (BPE) levels in peri-menopausal breast MRI compared with pre- and post-menopausal breast MRI.

**Methods:**

This study included 562 patients (55.8±12.3 years) who underwent contrast-enhanced dynamic breast MRI between 2011 and 2015 for clinical indications. We evaluated the BPE level, amount of fibroglandular tissue (FGT), and social and clinical variables. The inter-reader agreement for the amount of FGT and the BPE level was evaluated using interclass correlation coefficients. Associations between the BPE level and body mass index (BMI), ages of menarche and menopause, childbirth history, number of children, and the amount of FGT were determined using Spearman’s correlation coefficients or Mann-Whitney *U*-test. Pearson’s χ^2^ test was used to assess the difference in the frequency of BPE categories among the age-groups.

**Results:**

The inter-reader agreement was 0.864 for the amount of FGT and 0.840 for the BPE level, both indicating almost perfect agreement. The BPE level showed a weak positive correlation with the amount of FGT (Spearman’s ρ=0.271, *P*<0.001). BPE was not significantly correlated with BMI, childbirth history, number of births, or ages of menarche or menopause. BPE was greater in the peri-menopausal age-group compared with the corresponding pre- and post-menopausal age-groups, both with benign and malignant lesions.

**Conclusions:**

BPE was greater in the peri-menopausal stage than in the pre- and post-menopausal stages. Our results suggest that BPE showed a non-linear decrease with age and that the hormonal disbalance in the peri-menopausal period has a greater effect on the BPE level than was previously assumed.

## Highlights

• Factors influencing background parenchymal enhancement (BPE) on contrast-enhanced breast MRI are debated.
• BPE was greater in peri-menopause than in pre- and post-menopause in patients with benign and malignant lesions.
• BPE shows a convex rise in the peri-menopausal stage, rather than a linear decrease, through menopause.

## 1 Introduction

The degree of breast tissue enhancement on contrast-enhanced breast magnetic resonance imaging (MRI) is called background parenchymal enhancement (BPE). Although BPE was initially considered an incidental finding that could decrease breast MRI specificity and increase the frequency of biopsy recommendation [1], BPE is currently thought to have a mild impact on breast MRI interpretation [2]. Recent studies have suggested that BPE plays a role in predicting the risks of breast cancer [3], [4] and breast cancer recurrence [5], and in assessing the treatment response [6], [7] and prognosis [8].

BPE is highly influenced by endogenous and exogenous hormonal exposures that are related to the menopausal status [9], serum estrogen concentrations [10], and body mass index (BMI) [11], [12], as well as the use of menopausal hormone therapy [13], tamoxifen [14] and aromatase inhibitors [15].

The Stages of Reproductive Aging Workshop +10 staging system divides peri-menopause into three stages: *Stage −2* as an early menopausal transition with changes in the length of consecutive menstrual cycles by ≥7 days; *Stage −1* as a late menopausal transition with amenorrhea for ≥60 days; and *stage +1a* as early post-menopause with amenorrhea for 12 consecutive months [16]. The changes in reproductive hormones are most pronounced in the 2 years preceding and following the final menstrual period, overlapping with peri-menopause [17].

Some women experience symptoms as they transition to menopause, including vasomotor symptoms, breast tenderness, insomnia, migraines, and premenstrual dysphoria. However, the appearance and severity of symptoms vary considerably, and women experience different combinations of symptoms at different intensities [16]. BPE, which is affected by hormonal exposure, may also show drastic changes and fluctuate during the menopausal transition phase. However, the effect of hormonal disbalance during the peri-menopausal stage on BPE is unknown.

According to the European Society of Breast Cancer Specialists working group (EUSOMA), breast MRI is indicated in various cases, including screening patients at high risk of breast cancer, for pre-operative staging, and to resolve equivocal or discordant findings of conventional imaging [18]. Since daily MRI readings include a mixture of patients with benign and malignant lesions, it is important to know the nature of BPE in breasts with both benign and malignant lesions.

The aim of this study was to examine the possible change in BPE during the peri-menopausal stage compared with the pre- and post-menopausal stages, and to examine BPE in breasts with benign lesions and also those with malignant lesions.

## 2 Material and methods

### 2.1 Patient selection

In our institution, breast MRI examinations are performed for selected patients in accordance with national guidelines, which are in concordance with the EUSOMA recommendations [18]. The specific indications for breast MRI were as follows: 1) Screening of patients at high-risk for breast cancer; 2) Occult primary breast cancer; 3) Preoperative staging, whenever the exact tumour size or extension cannot be confidently identified with an expected impact on treatment decisions; 4) As a problem solving modality for equivocal or discordant findings at conventional imaging; 5) Evaluation of response to neoadjuvant chemotherapy; 6) Patients with pathological nipple discharge where galactography was technically unsuccessful and 7) Patients with Paget’s disease of the nipple before breast conserving surgery.

A total of 609 consecutive patients who underwent breast MRI at our university hospital between January 2011 and December 2015 were included in this retrospective study. For patients with at least two series of breast MRI, only the first contrast-enhanced dynamic examination was used in this analysis. Ten patients without contrast-enhanced dynamic MRI and 37 patients with bilateral breast cancer were excluded, leaving 562 eligible patients. No patients were undergoing chemotherapy or anti-hormonal therapy (e.g. tamoxifen) at the time of breast MRI.

Based on previous studies focusing on the peri-menopausal stage [19], [20], we defined the peri-menopausal stage as an age of 40—56 years, the pre-menopausal stage as an age of <40 years, and the post-menopausal stage as an age of >56 years.

Patient characteristics, including age, body mass index (BMI), childbirth history, ages at menarche and menopause, pathological information, and medication and surgical history were retrieved from the local digital archives.

The ethics committee of Kuopio University Hospital approved this study and waived the need for written informed consent due to the retrospective nature of the study. The study was conducted in accordance with the Declaration of Helsinki and followed all relevant national and international guidelines.

### 2.2 MRI protocol

The MRI protocol is summarised in Table 1. Breast MRI was performed using a 3.0 T MRI scanner (Philips Achieva TX, Philips N.V., Eindhoven, The Netherlands) with a dedicated seven-element phased-array bilateral breast coil.

**Table 1:** Breast magnetic resonance imaging protocol.

Sequence	TR/TE (ms)	In-plane Resolution (mm)	Slice Thickness (mm)	Scanning Time
T1-FFE	4.57 / 2.3	0.48 × 0.48	0.7	6 min 11 s
T2-TSE	5000 / 120	0.6 × 0.6	2	3 min 20 s
STIR	5000 / 60	1 × 1	2	5 min 40 s
T1 dynamic*	4.67 / 2.31	0.96 × 0.96	1	58.5 s
DWI #	7168 / 95	1.15 × 1.15	4	4 min 8 s

*eTHRIVE spectrally adiabatic inversion recovery (SPAIR) fat suppression; precontrast and six phases after the gadoterate meglumine injection (0.1 ml/kg, 3 ml/s) followed by a saline chaser.

# DWI: diffusion-weighted echo planar imaging with five respective b factors (0, 200, 400, 600, and 800 s/mm^2^) routinely performed after contrast administration. The apparent diffusion coefficients maps were automatically calculated linearly using the manufacturer’s method. FFE, fast field echo; TSE, turbo spin echo; STIR, short tau inversion recovery; TR, repetition time; TE, echo time

### 2.3 Image analysis

All images were independently and individually reviewed to determine the amount of fibroglandular tissue (FGT) and the BPE level by two specialised breast radiologists (HO, AI) with over 15 years of experience of reading breast MRI. Both radiologists were blinded to the patient information. The amount of FGT and the BPE level were visually assessed across the entire breast parenchyma according to the 5th edition of the Breast Imaging Reporting and Data System (BI-RADS)[21]. Fat-suppressed T1-weighted images were used to determine the amount of FGT. A combination of the pre- and post-contrast fat-suppressed T1-weighted and subtraction images was used to determine the BPE level. The amount of FGT was divided into four categories: a = almost entirely fat; b = scattered fibroglandular tissue; c = heterogeneous fibroglandular tissue, and d = extreme fibroglandular tissue. The BPE level was divided into four categories: 1 = minimal, 2 = mild, 3 = moderate, and 4 = marked.

In patients with a history of breast cancer or radiotherapy, data for the unaffected breast were included in the analyses.

In patients with benign findings where the BPE level differed between the breasts, the greater BPE was included in the analyses. Consensus was reached if there was disagreement between the two readers.

### 2.4 Statistical analysis

All statistical analyses were performed using SPSS for Windows version 29 (IBM Corporation, Armonk, NY, USA). Values of *P* < 0.05 were considered to be statistically significant.

The inter-reader (inter-rater) agreements for the amount of FGT and BPE level were evaluated by interclass correlation coefficients with a 95 % confidence interval (CI). An *r* value of 1.0 was considered as perfect agreement; 0.81–0.99 as almost perfect agreement; 0.61–0.80 as substantial agreement; 0.41–0.60 as moderate agreement; 0.21–0.40 as fair agreement; and ≤0.20 as slight agreement [22].

Differences in patient characteristics between those with malignant and benign lesions were assessed using the unpaired *t*-test or Pearson’s χ^2^ test. Associations between BPE and BMI, ages of menarche and menopause, childbirth history, number of births, and the amount of FGT were examined by Spearman’s correlation coefficient or the Mann-Whitney *U*-test. BMI and the ages of menarche and menopause were considered as continuous variables. The BPE level and the amount of FGT were considered as ordinal variables.

Pearson’s χ^2^ test was used to examine differences in the frequencies of BPE categories according to the menopausal age-group, divided into the pre-, peri-, and post-menopausal stages.

## 3 Results

The inter-reader agreement was 0.864 (95 % CI 0.819—0.896) for the amount of FGT, and 0.840 (95 % CI 0.808—0.866) for BPE, indicating almost perfect agreement in both cases.

The patient characteristics are presented in Table 2. Patients with benign lesions were younger and had higher BMI and greater BPE compared with patients with malignant lesions. However, there were no statistically significant differences in childbirth history, number of births, ages of menarche or menopause, or the amount of FGT between patients with benign and malignant lesions.

**Table 2:** Characteristics of patients divided into those with benign or malignant lesions.

	Benign	Malignant	Total	P value
Number	129	433	562	
Age	49.4 ± 13.7 (19−85)	57.7 ± 11.2 (21−87)	55.8 ± 12.3 (19−87)	< 0.001
BMI	26.1 ± 4.6 (16.4–41.0)	25.1 ± 4.1 (16.8–38.9)	25.3 ± 4.2 (16.4–41.0)	0.039
Childbirth history (number, %)				0.11
Yes	71 (55.0)	301 (69.5)	372(66.2)	
No	21 (16.3)	56 (12.9)	77(13.7)	
Unknown	37 (28.7)	76 (17.6)	113(20.1)	
Number of births	1.7 ± 1.5 (0−10)	1.8 ± 1.1 (0−6)	1.8 ± 1.2 (0−10)	0.69
Age of menarche	13.4 ± 1.4 (11−17)	13.3 ± 1.5 (9−19)	13.3 ± 1.5 (9−19)	0.71
Age of menopause	50.9 ± 3.2 (43−59)	50.6 ± 3.9 (39−61)	50.6 ± 3.8 (39−61)	0.64
Amount of FGT (number, %)				0.33
almost entirely fat	7 (5.4 %)	28 (6.5 %)	35 (6.2 %)	
scattered FGT	58 (45.0 %)	198 (45.7 %)	256 (45.6 %)	
heterogeneous FGT	51 (39.5 %)	183 (42.3 %)	41.6 (41.6 %)	
extreme FGT	13 (10.1 %)	24 (5.5 %)	37 (6.6 %)	
Level of BPE (number, %)				0.002
minimal	52 (40.3 %)	237 (54.7 %)	289 (51.4 %)	
mild	36 (27.9 %)	120 (27.7 %)	156 (27.8 %)	
moderate	29 (22.5 %)	59 (13.6 %)	88 (15.7 %)	
marked	12 (9.3 %)	17 (3.9 %)	29 (5.2 %)	

The data are shown as the mean ± standard deviation (range) unless otherwise noted.

The P-value indicates statistically significant differences in age, BMI, and BPE levels between patients with benign and malignant lesions. BMI, body mass index; BPE, background parenchymal enhancement; FGT, fibroglandular tissue

Table 3 shows the associations between BPE levels and variables that may influence BPE, such as BMI, gynaecological history, and the amount of FGT. The BPE level showed a weak positive correlation with the amount of FGT (Spearman’s ρ = 0.271, *P* < 0.001). The BPE level was not significantly correlated with BMI, childbirth history, number of births, or ages of menarche or menopause.

**Table 3:** Associations between background parenchymal enhancement (BPE) levels and variables that may influence BPE, such as BMI, gynaecological history, and the amount of FGT.

	P value
BMI	0.689
Childbirth history	0.189
Number of births	0.221
Age of menarche	0.065
Age of menopause	0.472
Amount of FGT	<0.001

BMI, body mass index; BPE, background parenchymal enhancement; FGT, fibroglandular tissue

**Fig. 1:**
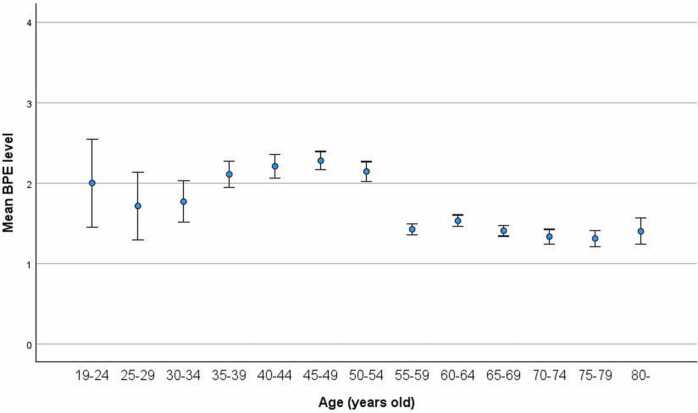
Age-related changes in BPE levels indicate that the BPE level remains high during the pre-menopausal stage, but the peak occurs during the peri-menopausal stage, and the BPE then consistently declines after menopause. Age was divided into 5-year increments, and the mean BPE level in each age-group was plotted with standard error bars. BPE, background parenchymal enhancement.

The changes in BPE with age are shown in Fig. 1. Although younger age groups generally exhibited relatively high BPE levels despite a larger standard error, the graph shows that the BPE level reached a gentle peak at the age-group of 45—49 years, followed by the age-groups of 40—44 and 50—54 years. After the age-group of 55–59, the BPE level dropped significantly and then remained relatively stable. The BPE level remained high during the pre-menopausal stage, but the peak occurred during the peri-menopausal stage and consistently declined after menopause.

The association between BPE and each menopausal age-group is summarised in Table 4. The association between BPE and menopausal age-group was significant for the benign (χ^2^ = 17.7, *P* < 0.01), malignant (χ^2^ = 67.2, *P* < 0.01), and overall (χ^2^ = 88.0, *P* < 0.01) groups of patients. In the benign peri-menopausal age-group, the observed count for the minimal BPE category was significantly lower than the expected count, while the observed counts for the moderate and marked BPE categories were significantly higher than expected. This indicates that BPE level in the benign peri-menopausal age-group was skewed towards being higher than statistically expected. Similarly, in the benign post-menopausal age-group, the observed count for the minimal BPE category was higher than expected, while the marked BPE category was lower. In patients with malignant lesions, the peri-menopausal age-group also showed a higher proportion of moderate and marked BPE categories compared to the pre- and post-menopausal age-groups, while the minimal and mild BPE categories were fewer. Overall, BPE was elevated in the benign and malignant peri-menopausal age-groups compared with the corresponding pre- and post-menopausal age-groups.

**Table 4:** Associations between BPE levels and menopausal age-groups in patients with benign or malignant lesions, and the overall cohort.

(a) Patients with benign lesions					
		Menopausal age-group			
		Pre (<40)	Peri (40−56)	Post (56<)	Total
BPE	minimal	13 (41.9 %)	19 (30.6 %)	20 (55.6 %)	52 (40.3 %)
		0.2	−2.2	2.2	
	mild	11 (35.5 %)	13 (21.0 %)	12 (33.3 %)	36 (27.9 %)
		1.1	−1.7	0.9	
	moderate	4 (12.9 %)	21 (33.9 %)	4 (11.1 %)	29 (22.5 %)
		−1.5	3.0	−1.9	
	marked	3 (9.7 %)	9 (14.5 %)	0 (0.0 %)	12 (9.3 %)
		0.1	2.0	−2.3	
(b) Patients with malignant lesions					
		Menopausal age-group			
		Pre (<40)	Peri (40−56)	Post (56<)	Total
BPE	minimal	5 (23.8 %)	70 (40.9 %)	162 (67.2 %)	237 (54.7 %)
		−2.9	−4.7	5.8	
	mild	11 (52.4 %)	44 (25.7 %)	65 (27.0 %)	120 (27.7 %)
		2.6	−0.7	−0.4	
	moderate	3 (14.3 %)	43 (25.1 %)	13 (5.4 %)	59 (13.6 %)
		0.1	5.6	−5.6	
	marked	2 (9.5 %)	14 (8.2 %)	1 (0.4 %)	17 (3.9 %)
		1.4	3.7	−4.2	
(c) All patients					
		Menopausal age-group			
		Pre (<40)	Peri (40−56)	Post (56<)	Total
BPE	minimal	18 (34.6 %)	89 (38.2 %)	182 (65.7 %)	289 (51.4 %)
		−2.5	−5.3	6.7	
	mild	22 (42.3 %)	57 (24.5 %)	77 (27.8 %)	156 (27.8 %)
		2.5	−1.5	0.0	
	moderate	7 (13.5 %)	64 (27.5 %)	17 (6.1 %)	88 (15.7 %)
		−0.5	6.5	−6.1	
	marked	5 (9.6 %)	23 (9.9 %)	1 (0.4 %)	29 (5.2 %)
		1.5	4.2	−5.1	

The data are shown as the count (percentage) in the upper row and the adjusted residual in the lower row.

BPE, background parenchymal enhancement; Pre, pre-menopause; Peri, peri-menopause; Post, post-menopause

## 4 Discussion

We investigated the BPE levels on contrast-enhanced breast MRI in the peri-menopausal stage compared with the pre- and post-menopausal stages in patients with benign lesions and also those with malignant lesions. This study is the first to demonstrate that BPE is more prominent in the peri-menopausal stage than in the pre- and post-menopausal stages, and this was apparent in patients with benign lesions as well as those with malignant lesions.

Reproductive aging is associated with elevated levels of follicle stimulating hormone and luteinising hormone, and reduced levels of oestradiol and progesterone during the peri-menopausal stage [23]. Menopausal symptomology varies among ethnic groups, cultures, socioeconomic groups, and climates, and the timing and degree of hormonal changes vary considerably among individuals [16]. Thus, the internal changes that occur during the menopausal transition may contribute to the elevated BPE in the peri-menopausal stage. Our results suggest a significant effect of the hormonal disbalance during the peri-menopausal stage on BPE, which is visible on MRI. In other words, BPE is potentially influenced by a wider variety of factors than those identified to date.

In line with previous studies [24], [25], our findings confirm that the BPE level is positively correlated with the amount of FGT, although the correlation was relatively weak in the present study. The level of BPE and amount of FGT may reflect the normal proliferation of breast epithelial cells, which is sensitive to hormonal fluctuations [26]. This may be depicted by breast MRI, where greater BPE is associated with denser FGT and lower BPE with sparser FGT. The variation in the strength of the correlation between BPE levels and the amount of FGT may be attributed to the absence of systematic methods for quantifying BPE and breast density in studies related to these topics over time. This underscores the necessity for a universally accepted, objective approach to reliably assess both BPE levels and the amount of FGT. Furthermore, we found that the BPE level was lower in the post-menopausal age-group than in the pre- and peri-menopausal groups, which is consistent with a previous report [9]. Importantly, the BPE level proved to be greater in the peri-menopausal age-group, even compared with the younger pre-menopausal age-group, which is not indicated in previous literature, making our study the first to demonstrate this. In other words, BPE does not show a linear decrease during the transition from pre-menopause to post-menopause, but instead shows a peak in the peri-menopausal period and then declines after menopause.

Watt et al. from the IMAGINE Study group conducted a case-control investigation of BPE in a cohort of 1476 women, comprising participants from both high-risk screening and diagnostic evaluations [27]. The subjects were categorized as premenopausal or postmenopausal, revealing a monotonic increase in breast cancer odds across the quartiles of BPE. Our findings indicate that BPE levels correlate with menopausal stages and the amount of FGT. In contrast, Watt et al. reported that the relationship between breast cancer odds and BPE extent did not significantly vary based on menopausal status or FGT volume. Although our study did not specifically assess the link between BPE and breast cancer risk, it underscores the importance of further investigating the impact of BPE on breast cancer risk, particularly concerning menopausal stages, including the peri-menopausal phase.

The inter-reader agreement was almost perfect for both the amount of FGT and the BPE level. Compared with previous studies, which reported fair to moderate inter-observer agreement [28], [29], our results can be explained by the readers’ extensive experience of performing breast MRI.

We observed no significant correlation between the BPE level and BMI, whereas prior studies reported a positive correlation [11], [12]. Brown et al. proposed that metabolic alterations associated with obesity, including insulin resistance, elevated leptin levels, and decreased adiponectin levels, may be critical determinants of the relationship between obesity and BPE [11]. On the other hand, Hellgren et al. suggested that the increased levels of BPE observed in obesity may stem from an inflammatory environment in breast tissue induced by obese adipocytes [12]. Our findings suggest a potential discrepancy with previous research on these metabolic factors, which might reflect differences in inclusion criteria among the studies. That is, Brown et al. utilised relatively strict patient eligibility criteria, as they only included pre-menopausal women aged 18–50 years at high risk of breast cancer, with some conditions on diet and exercise [11]. In contrast, our study included pre-, peri-, and post-menopausal women with a diverse range of breast cancer risks and imposed no restrictions on diet or exercise. Similarly, Hellgren et al. only included healthy participants aged ≥43 years from a screening program [12], whereas our study incorporated both screening participants and clinical patients, encompassing individuals with both benign and malignant lesions.

None of the other measured parameters, including childbirth history, number of births, and the ages of menarche or menopause, were correlated with the BPE level, although they are known to influence the risk of breast cancer [30]. Of particular note, we found that the age at menopause was not correlated with BPE. Many factors are known to affect the age of menopause, including the mother’s age at menopause, the age at menarche, gestational age, use of oral contraceptives, irregular menstrual cycle, number of pregnancies, BMI, smoking, alcohol consumption, physical activity, unilateral oophorectomy, serum lead levels, consumption of polyunsaturated fat, socioeconomic status, and education level [31]. Although menopause itself is a significant life event that may be a turning point for the change in BPE, the BPE level was not significantly influenced by age at menopause. Although our analysis did not reveal significant correlations between BPE levels and reproductive history, such as childbirth history, number of births, and the ages of menarche and menopause, the intricacies of reproductive processes necessitate more rigorous investigations focused specifically on these aspects. Research employing artificial intelligence may yield valuable insights into these relationships.

To enable personalised care of breast cancer with optimal methods, it is important to understand the characteristics of BPE, which is a promising prognostic imaging marker for the risk of breast cancer and treatment outcomes. Nevertheless, several factors might confound the interpretation of BPE levels. First, the qualitative assessment of BPE is subjective and may have limited reliability because of inter-reader variability. However, the inter-reader agreement could be improved by training breast radiologists. In comparison, quantitative evaluation is more objective and yields greater consistency, but the methods have not been standardised or adopted in clinical practice [27,32]. More studies are needed to identify reliable and reproducible methods for quantitative BPE assessment. Second, because BPE fluctuates with the menstrual cycle, peaking in weeks 3 and 4 and declining in week 2, the optimal timing of breast MRI is thought to be between days 7 and 14 of the menstrual cycle [32]. Although screening is usually scheduled according to the individual’s menstrual cycle, this is not always possible for urgent cases requiring prompt diagnosis and treatment, which is common in clinical practice. From the perspective of BPE, the optimal timing of imaging remains unclear.

A variety of symptoms may be experienced during the peri-menopausal period, including breast tenderness, and these symptoms fluctuate over time and vary between individuals. Although the general relationship between BPE and age has been studied, to our knowledge, no research has focused on the changes in BPE during the peri-menopausal stage or the correlation between the severity of clinical symptoms and BPE. Our study has shown that BPE fluctuates and reaches a peak during the peri-menopausal stage, rather than decreasing linearly over time. To more accurately understand the mechanisms underlying the changes in BPE, future studies should continue to explore the significance of the changes that occur during the peri-menopausal stage.

This study was limited by its retrospective design and the fact that the data were obtained from routine clinical practice rather than specifically collected cohorts for research purposes. Consequently, there is a lack of precise information on endocrine hormonal changes and menstrual cycle characteristics, such as regularity and frequency, which may lead to misclassification of the menopausal stage. Although most patients were adequately managed in terms of imaging timing relative to their menstrual cycles, some could not follow the optimal schedule for clinical reasons. Our findings indicate the need for large-scale prospective studies with precise menopausal staging that include a broad range of participants from various ethnicities and ages, while also considering other variables that might affect BPE. Furthermore, future research implementing medical interaction systems and artificial intelligence [33] should prioritize stringent control of these variables to more effectively isolate the effects of menopausal stage on BPE. Nevertheless, this is the first study to investigate the change in BPE, with a particular focus on the peri-menopausal stage, and all consecutive patients were included in this study to reduce bias.

In summary, our results show a prominent increase in BPE levels during the peri-menopausal stage compared with the pre- and post-menopausal stages. This increase in BPE was found in patients with benign lesions as well as those with malignant lesions.

## Ethical statement

The ethics committee of Kuopio University Hospital approved this study and waived the need for written informed consent due to the retrospective nature of the study. The study was conducted in accordance with the Declaration of Helsinki and followed all relevant national and international guidelines.

## CRediT authorship contribution statement

**Aki Nykänen:** Writing – review & editing, Data curation. **Juhana Hakumäki:** Writing – review & editing, Supervision, Funding acquisition. **Ritva Vanninen:** Writing – review & editing, Validation, Supervision, Project administration, Funding acquisition. **Mazen Sudah:** Writing – review & editing, Validation, Supervision, Resources, Project administration, Methodology. **Amro Masarwah:** Writing – review & editing, Data curation. **Aleksandr Istomin:** Writing – review & editing, Data curation. **Hidemi Okuma:** Writing – original draft, Methodology, Investigation, Funding acquisition, Formal analysis, Data curation, Conceptualization.

## Declaration of Competing Interest

The authors declare that they have no known competing financial interests or personal relationships that could have appeared to influence the work reported in this paper.
